# The nature of phase and amplitude modulation in heterodyne background-oriented schlieren systems

**DOI:** 10.1007/s00348-026-04289-w

**Published:** 2026-08-25

**Authors:** Sami Tasmany, Robert Kuschmierz, Jakob Woisetschläger

**Affiliations:** 1 Institute of Thermal Turbomachinery and Machine Dynamics, Graz University of Technology, Inffeldgasse, 25, 8010 Graz, Styria Austria; 2 Institute of Solid-State Electronics, Dresden University of Technology, Helmholtzstraße 10, 01069 Dresden, Germany

## Abstract

Heterodyne background-oriented schlieren is examined here as a diagnostic that separates phase and amplitude effects in reactive flows, and it is applied to a lean premixed swirl-stabilised methane flame. In this approach, a periodic fringe background and Fourier demodulation yield line-of-sight displacement fields associated with refractive-index gradients, while the fringe amplitude modulation isolates intensity redistribution in a manner that is mathematically decoupled from phase. Building on this separation, ray-tracing analysis clarifies how the optical system and the refractive-index field jointly shape the recorded image, thereby revealing the physical origin of the fringe amplitude modulation signal. In particular, the fringe amplitude modulation field is shown to behave similarly to a focussed shadowgraph that preferentially amplifies high-gradient, small-scale features linked to combustion and suppresses low-curvature convection features. This selectivity is governed by the acquisition aperture through depth-of-focus and aperture-related ray blockage. Finally, controlled experiments with extensive image acquisition corroborate these interpretations and provide practical guidance on carrier-filtering and acquisition settings to avoid spectral overlap and maintain accuracy.

**Graphical abstract:**

**Figure:**
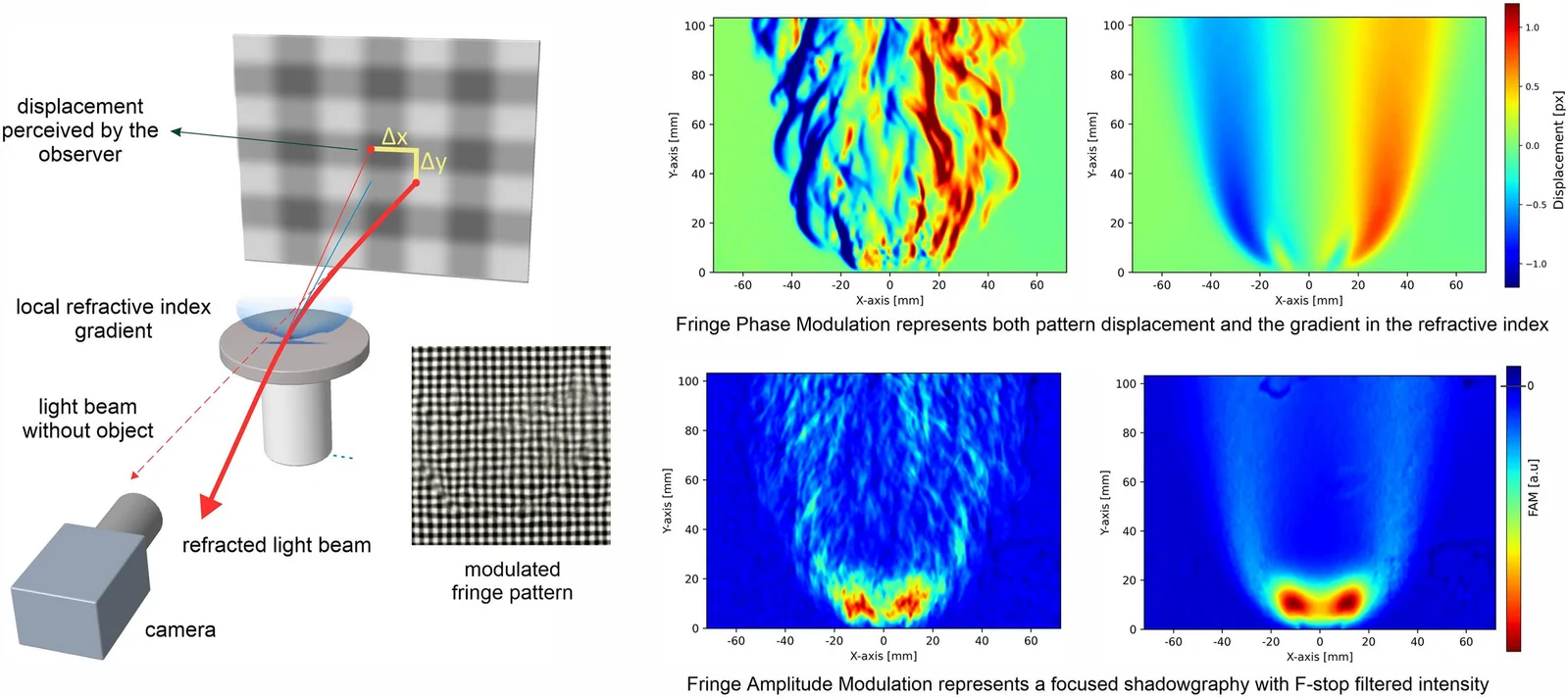

**Supplementary Information:**

The online version contains supplementary material available at https://doi.org/10.1007/s00348-026-04289-w.

## Introduction

Ever since Mach’s and Salcher's pioneering visualisation of fast-moving projectiles in 1887 (Mach and Salcher 1887), the schlieren method has provided a straightforward means of visualising density fluctuations in gas flows, particularly in flames (Settles 2001). In recent decades, the background-oriented schlieren (BOS) method has become established for experimental investigations, as it allows for computer-based analysis of the images. When using BOS as outlined in the study by Schmidt et al. (2025), the refractive index and density distributions are determined by correlating the displacement of background structures caused by the deflection of light rays in the refractive-index field of a fluid flow. BOS has proved to be a successful tool for measuring local refractive-index gradients in flames (Martins et al. 2023; Gao et al. 2023) as well as reference free shadowgraphy (Gardner et al. 2020). As any displacement of background structures can be used for measurement in BOS, several authors have conducted detailed investigations of periodic fringe patterns or grids in combination with demodulation via Fourier transformation using synthetic data (Blanco et al. 2016a, b; Wildeman 2018; Vinnichenko et al. 2023). Approaches using Fourier analysis have been shown to increase sensitivity and are significantly faster than the conventional use of correlation algorithms for speckled backgrounds (Wildeman 2018).

In a recent publication, the authors present a method that combines the advantages of BOS with those of heterodyne holographic interferometry (HHI), a technique that has been used successfully for high-resolution interferometry for many decades (Takeda 1990; Kreis 1996; Hipp et al. 2004). This variant of BOS is referred to by Tasmany et al. (2024) as 'Heterodyne Background-Oriented Schlieren' (HBOS) and is used to investigate thermoacoustic oscillations. The implementation of HHI methods in BOS facilitates the accuracy of fringe shifts within the millifringe range, in addition to the monitoring of phase modulation and amplitude modulation of the carrier fringes heterodyned. Standardised HBOS workflows are also advantageous in that they allow for the compensation of disturbances in the optical beam path and thus in the background pattern. To that end, a comparison is made of the periodic background pattern with and without an object. The causes of such disturbances are varied and may, for example, lie in factors such as density variations in the hot glass or aberrations in the imaging process. Further details on this workflow can be found in the following section.

**Fig. 1:**
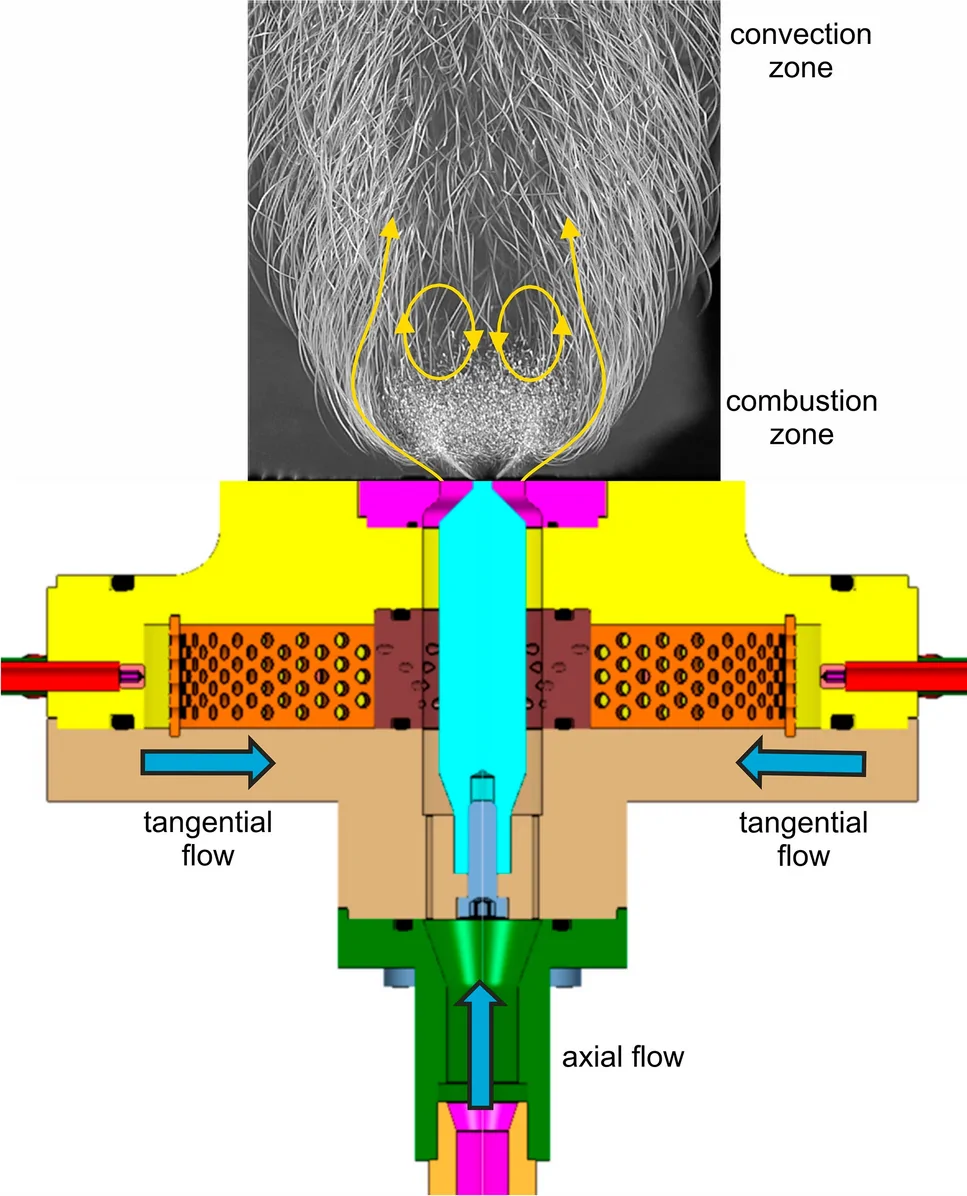
Concept of the swirl-stabilised flame used in this work. This type of flame is commonly employed in aero-engines and ground-based gas turbines. The image in the background comprises an average over 700 schlieren recordings, with only the brightest gradients having been superimposed. The tangential swirl in the inflow causes the flow to expand, resulting in a backflow of hot exhaust gas in the centre. This, in turn, ignites the fresh gas mixture. The image clearly shows the highly turbulent combustion zone

This study focusses on the application of HBOS in a swirl-stabilised flame, and discusses the nature of the fringe phase and amplitude modulation associated with the refractive-index field in this type of flame. The concept of a swirl-stabilised flame, as commonly used in aircraft engines and ground-based gas turbines, is illustrated in Fig. 1. The image in the background of this figure consists of an average over 700 schlieren images, with only the brightest gradients superimposed. The presence of tangential velocity in the inflow region causes a swirl and subsequent expansion of the flow. This results in a backflow of hot exhaust gas in the centre of the flame. In this type of detached flame, the hot exhaust gas in the recirculation zone ignites the fresh gas mixture. The image clearly shows the highly turbulent combustion zone above the orifice of the burner.

In the context of lean combustion, this type of flame has been observed to be susceptible to thermoacoustic instabilities (Dowling and Mahmoudi 2015). However, these instabilities do not only have disadvantages. In the instance of lean hydrogen combustion, it has been demonstrated that the strategic utilisation of the interaction between acoustic modes and reactive flow can result in a reduction in nitrogen oxides (Paulitsch et al. 2023). In order to facilitate a thorough investigation of these thermoacoustic instabilities, it is essential to examine the heat release and its fluctuations in this type of flame.

The objective of the investigations conducted here is to discuss the nature of the phase and amplitude modulation of the HBOS background fringe system in terms of its usefulness in the study of thermoacoustic oscillations in turbulent swirl-stabilised flames.

## Materials and methods

### Experimental set-up

The German word 'schliere' refers to a streaky area in translucent materials characterised by slight variations in density or refractive index. However, as it causes very little light deflection, a schliere is difficult to see with the naked eye.

**Fig. 2:**
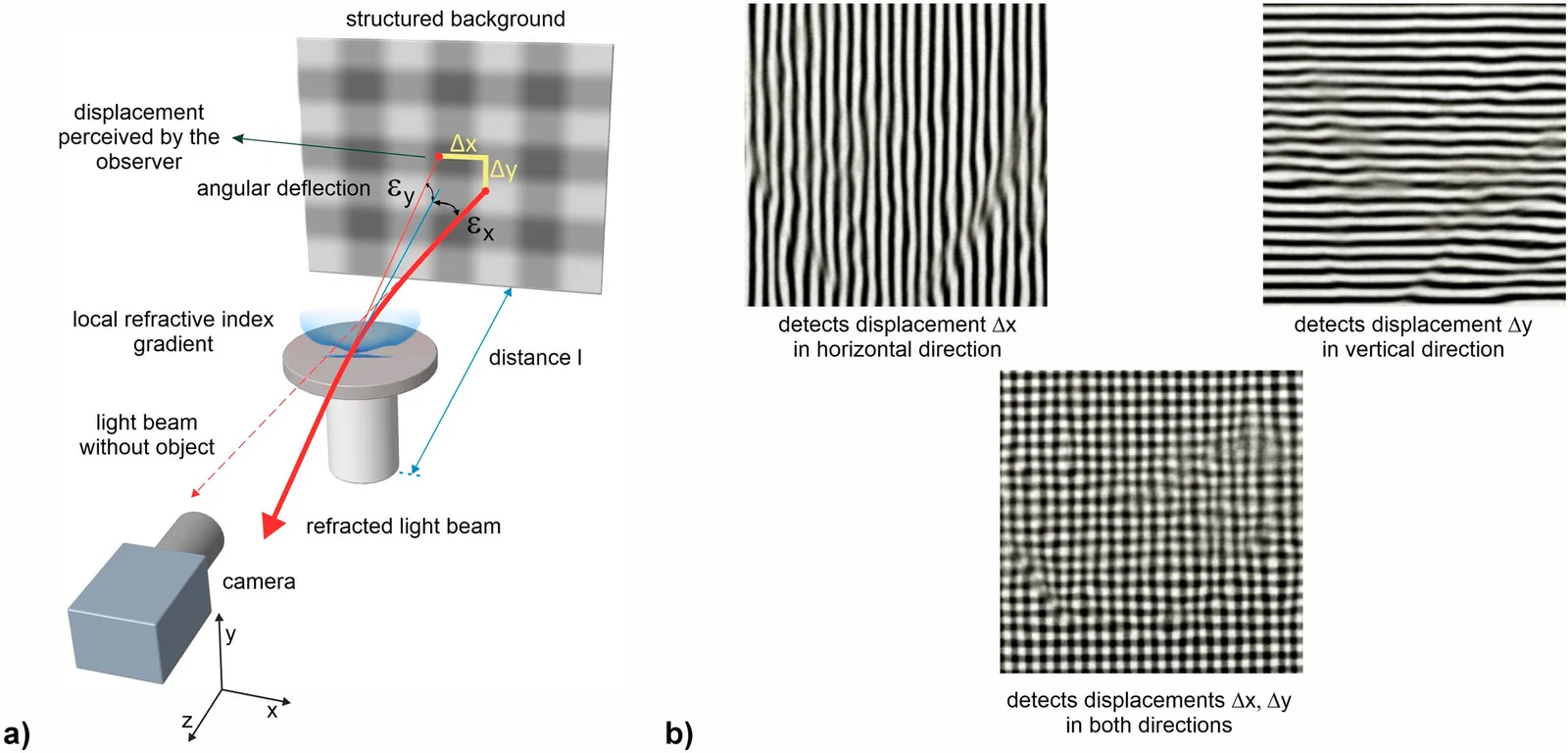
Schematic illustration of the experimental set-up. Section **a** explains the background displacement perceived by the observer when the light beam is deflected by the flame's refractive-index gradient. Section **b** shows how the fringe patterns captured by the camera are displaced by the refractive-index gradients in the flame

Figure 2a shows a schematic illustration of the experimental set-up. A grid-like pattern with fringes 1 mm wide was printed on matte tracing paper (RTL49 A3, Altiel Ltd, Buckinghamshire, UK) to serve as the background pattern. This pattern was placed on a 420 mm × 297 mm glass plate backlit using a set of DC LED lamps (120 W LED work lights, 12,000 lm, B&S Warenhandels GmbH, Achim, Germany). The lamps were powered by a 650 W power supply (SSP-650RS, Sea Sonic Electronics Co. Ltd., Taipei, Taiwan). The distance l between the background screen and the burner was 0.5 m, as was the distance between the burner and the camera. All images were captured using a GigE monochrome acA140-73gm camera (Basler AG, Ahrensburg, Germany). This camera was fitted with a 12 mm focal length lens (C231224-5M-P, Basler AG, Ahrensburg, Germany) and was focussed on the background pattern. Figure 2a illustrates how light emitted from the screen is deflected by the local refractive-index gradient present in a flame. Assuming that the beam is deflected by these refractive-index gradients by a very small amount, the equations of geometric optics can be linearised. For the observed displacement *Δx* and *Δy* of the background pattern, this yields:

1a $$\varDelta x = l \;\tan \varepsilon_{x} = l\int_{D} {\frac{1}{n}\;\frac{\partial n}{{\partial x}}\;{\mathrm{d}}z}$$

1b $$\varDelta y = l\; \tan \varepsilon_{y} = l\int_{D} {\frac{1}{n}\;\frac{\partial n}{{\partial y}}\;{\mathrm{d}}z}$$

Here, *Δx* and *Δy* are the displacements of the background pattern perceived by the observer along the *x*- and *y*-axes, respectively. *ε*_*x*_ and *ε*_*y*_ are the deflection angles of the light beam, and *n* is the local refractive index. The *z*-axis is oriented along the line of sight (LOS), with *D* denoting the optical path length through the flame. As demonstrated in Fig. 2b, the fringe pattern captured by the camera is displaced by the refractive-index gradients in the flame, and this is perceived as a modulation of the otherwise uniform carrier fringes.

At the same time, this slight phase distortion of the wavefront within the refractive-index gradient in the flame causes a redistribution of light intensity on the screen. Due to the light irradiance redistribution caused by the flame's refractive-index gradient distribution, certain points on the screen appear darker, while others appear brighter. This effect is known as shadowgraphy and causes fringe blurring or a shadow on the screen. Given the assumption that the beam deflection within the object is negligible, the change in irradiance at individual points on the screen can be expressed as a linear relationship based on the relative change in total intensities *I* (with flame) and *I’* (without flame, *I’* > 0 for all positions), as follows:

2 $$\frac{{I - I^{\prime } }}{{I^{\prime } }} = \frac{\Delta I}{{I^{\prime } }} = l\int_{D} {\left( {\frac{1}{n}\frac{{\partial^{2} n}}{{\partial x^{2} }} + \frac{1}{n}\frac{{\partial^{2} n}}{{\partial y^{2} }}} \right){\mathrm{d}}z}$$

Heuristic and analytical derivations of Eqs. 1a, 1b and 2 can be found in the supplement to this manuscript. Discussions of these derivations can also be found in Weyl (1954), Merzkirch (1987), Oertel and Oertel (1989), and Settles (2001).

The experiment was conducted testing an open swirl-stabilised flame that operated with methane at ambient pressure and 8.00 bar supply pressure. The supply pressure of air was 6.25 bar. These gases were delivered to the test facility through two separate lines. The mass flows of the two gases were measured using two ELFLOW Select flowmeters (Bronkhorst High-Tech B.V., Ruurlo, the Netherlands) and recorded as 0.074 g/s for methane and 1.320 g/s for air, resulting in a simplified geometric swirl number of 0.53 as calculated by Greiffenhagen et al. (2019). The gas flows were premixed and subsequently divided into tangential and axial flows (cmp. Fig. 1). Following ignition, this generates a flame of approximately 3.4 kW. Additionally, a siren was present to introduce a pressure fluctuation along the axial feeding line (Giuliani et al. 2020). For a more detailed description of the experimental set-up, please refer to the document by Tasmany et al. (2024).

### Heterodyne background-oriented schlieren technique

HBOS uses a combination of two well-established techniques: the BOS method (Schmidt et al. 2025) and the HHI technique (Kreis 1996). In the HBOS approach, the speckled background pattern typically employed in the BOS method is replaced with a one- or two-dimensional fringe pattern, as shown in Fig. 2b. As with the HHI technique, the fringe pattern serves as a spatial carrier frequency, which becomes distorted (modulated in phase) due to the deflection of light rays at the refractive-index gradient. At the same time, local amplitude modulation occurs. The phase and amplitude of a one-dimensional fringe pattern can be described by the following equation:

3 $$i\left( x \right) = i_{0} \left( x \right) + a\left( x \right){\mathrm{cos}}\left[ {\varphi_{{{\mathrm{Carrier}}}} \left( x \right) + \varphi_{{{\mathrm{Displacement}}}} \left( x \right)} \right]$$

**Fig. 3:**
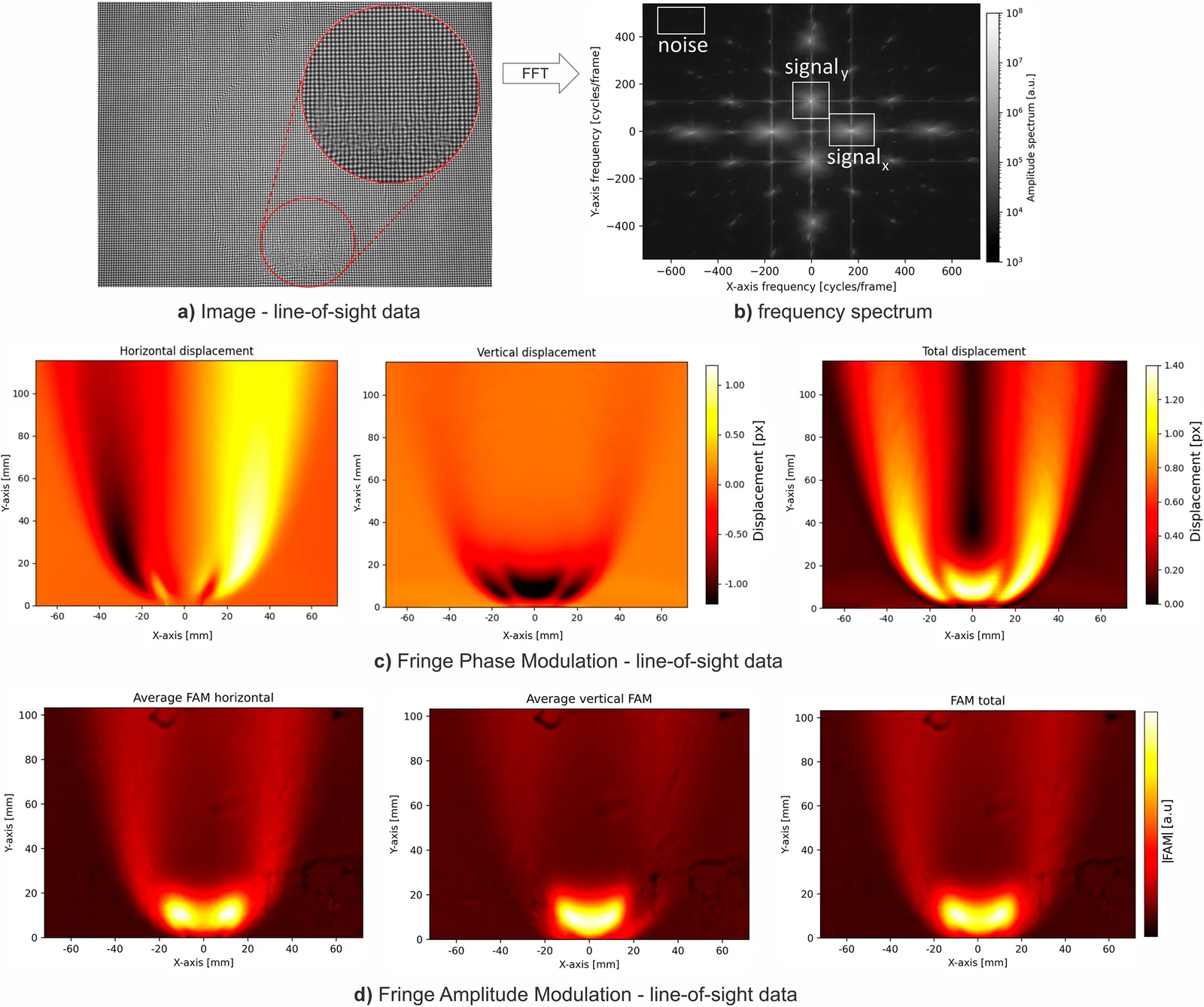
Visual representation of the workflow for fringe analysis. The first step in the process is to Fourier transform the image. In a second step, the spectrum of the image is filtered. In a last step, the fringe phase modulation (fringe displacements) and the fringe amplitude modulation (FAM) can be calculated from the filtered back transformation

In this equation, *i*_0_(*x*) represents the low-frequency background intensity noise, such as that caused by uneven illumination, *a*(*x*) represents the fringe amplitude*, φ*_Carrier_(*x*) is the linear phase related to the carrier-fringe system spatially heterodyned, and *φ*_Displacement_(*x*) denotes the phase related to the distortions of the carrier-fringe system in *x*-axis direction caused by the beam deflection in the refractive-index gradient field. Figure 3a provides an example of such a background pattern. This instantaneous recording shows how the beam deflection caused by the refractive-index gradients in the flame leads to modulation of the carrier frequency in two dimensions. It is possible to extract the signal in Eq. 4 by applying Euler’s identity to Eq. 3.

4 $$g\left( x \right) = \frac{1}{2}a\left( x \right){\mathrm{e}}^{{j\left[ {\varphi_{{{\mathrm{Carrier}}}} \left( x \right) + \varphi_{{{\mathrm{Displacement}}}} \left( x \right)} \right]}}$$

The workflow for this type of fringe analysis is illustrated in Fig. 3 and discussed in detail by Kreis (1996), Hipp et al. (2004) and Tasmany et al. (2024). Due to the severe turbulence of the combustion process, 10,000 individual frames were recorded in this study in order to obtain an average gradient of refractive-index value with a high confidence and signal-to-noise ratio (SNR). At the conclusion of the recording process with flame, a set of reference images is obtained without the flame. In the initial analysis step, a two-dimensional Fourier transform is performed on each individual image. These Fourier spectra are then bandpass-filtered around the carrier frequency (Fig. 3b). The SNR is estimated based on the bandpass-filtered signal region compared to a region in the spectrum that is distant from the zero order and the carrier frequency. The next step in the procedure is to transform the bandpass-filtered signals back into the image domain via inverse Fourier transformation. This results in the reconstruction of the locally resolved phase and amplitude distribution of the fringe pattern. Due to the properties of the arctangent function, this phase is modulo 2π and must be unwrapped at the discontinuities. The resulting phase map shows a predominantly linear phase distribution of the carrier fringes, in the case of the recordings with flame, modulated by the required phase *φ*_Displacement_(x).

5a $$\varphi_{{{\mathrm{Carrier}}}} \left( x \right) + \varphi_{{{\mathrm{Displacement}}}} \left( x \right) = {\mathrm{arctan}}\frac{{{\mathrm{Im}}\left| {g_{{{\mathrm{filtered}}}} \left( x \right)} \right|}}{{{\mathrm{Re}}\left| {g_{{{\mathrm{filtered}}}} \left( x \right)} \right|}}$$

5b $$\varphi_{{{\mathrm{Carrier}}}} \left( x \right){\prime} = {\mathrm{arctan}}\frac{{{\mathrm{Im}}\left| {g_{{{\mathrm{filtered}}}} \left( x \right)^{\prime} } \right|}}{{{\mathrm{Re}}\left| {g_{{{\mathrm{filtered}}}} \left( x \right)^{\prime} } \right|}}$$

Equation 5a defines the modulated phase with the flame, starting from the filtered spectrum, while Eq. 5b describes the reference without a flame. *φ*_Displacement_(*x*) is obtained by subtracting the two. The prime symbol denotes the background image that was recorded without flame. To obtain a displacement in pixels from the phase *φ*_Displacement_(*x*), it is necessary to determine the local period *λ*(*x*) of the carrier-fringe system in pixels. It is important to note that the period may vary locally due to the presence of curved windows. This workflow enables the calculation of the line-of-sight (LOS) displacements *Δx* and *Δy* of the background pattern from Eqs. 1a, 1b, which can then be related to the refractive-index gradients.

6a $$\Delta x = \varphi_{{{\mathrm{Displacement}}}} \left( x \right)\frac{\lambda \left( x \right)}{{2\pi }}$$

6b $$\Delta y = \varphi_{{{\mathrm{Displacement}}}} \left( y \right)\frac{\lambda \left( y \right)}{{2\pi }}$$

It is possible to add these two components, *Δx* and *Δy*, in a vectorial way. The total magnitude of the displacement can be obtained by using the Euclidean norm, as shown in the plots presented in Fig. 3c.

On the other hand, the fringe amplitudes *a*(*x*) and *a*(*y*) can be derived from the filtered signal by:

7a $$a_{x} \left( x \right) = \sqrt {{\mathrm{Re}} \left| {g_{{{\mathrm{filtered}}}} \left( x \right)} \right|^{2} + {\mathrm{Im}} \left| {g_{{{\mathrm{filtered}}}} \left( x \right)} \right|^{2} }$$

7b $$a_{y} \left( y \right) = \sqrt {{\mathrm{Re}} \left| {g_{{{\mathrm{filtered}}}} \left( y \right)} \right|^{2} + {\mathrm{Im}} \left| {g_{{{\mathrm{filtered}}}} \left( y \right)} \right|^{2} }$$

The total amplitude *a*(*x,y*) is once again calculated by applying the Euclidean norm. In the following discussion of the results, we define the fringe amplitude modulation (FAM) as follows:

8a $${\mathrm{FAM}}_{x} \left( x \right) = \frac{{a_{x}^{2} \left( x \right) - a_{x}^{2} \left( x \right)^{\prime} }}{{a_{x}^{2} \left( x \right)^{\prime} }}$$

8b $${\mathrm{FAM}}_{y} \left( y \right) = \frac{{a_{y}^{2} \left( y \right) - a_{y}^{2} \left( y \right)^{\prime} }}{{a_{y}^{2} \left( y \right)^{\prime} }}$$

As in Eq. 5a, 5b, the prime denotes the background image that was recorded without flame. The squared fringe amplitude is used to emphasise its direct relationship with optical intensity, as defined in shadowgraphy (compare Eq. 26 in the supplement).

Since this method is a LOS method, tomographic algorithms must be applied to reconstruct local distributions (Tasmany et al. 2025a, b; Gao et al. 2023; Martins et al. 2023). However, the integration of fluctuations along the line of sight can lead to an incorrect weighting of high-frequency fluctuations. For a more detailed analysis of this type of uncertainty, please refer to Mayrhofer and Woisetschläger (2001). Finally, the relationship between the refractive index and density is determined by the Gladstone–Dale number, with the values for combustion gases documented in Gardiner et al. (1981).

### Practical conditions for phase and amplitude modulation separation

The separation between phase modulation and fringe amplitude modulation holds under the following practical conditions. First, in the Fourier spectra the carrier frequency signal and its modulation remain clearly separated from the zero order and from higher harmonics; this is achieved by selecting a suitable carrier-fringe frequency and image sampling, and by applying the same bandpass window to flame and reference data. Second, the imaging response remains linear, sensor saturation is avoided, and the aperture is chosen so that aberration due to the finite circle of confusion (Hecht 2002) do not smear the pattern at scales comparable to the carrier period to the point of making it undetectable. Third, the modulation of the carrier frequency is small to moderate relative to the carrier in the instantaneous recordings. Under these conditions, the phase modulation yields (LOS) displacements linked to refractive-index gradients, while the FAM isolates a response similar to focussed shadowgraphy through small-scale aberration and is independent of the phase. If these conditions are not met, for example, if the carrier and the first- or second-order components begin to overlap in the spectra, or if the result becomes strongly dependent on the chosen bandpass, phase modulation becomes unreliable and the FAM should be treated as qualitative in the affected regions.

### Ray tracing

In order to gain a better comprehension of the nature of the HBOS output, a series of ray-tracing simulations has been performed. The ray-tracing algorithm serves in particular to elucidate the physical meaning of the FAM field.

In ray tracing, a bundle of light ray is emitted from a background and propagates through a physical domain until it reaches the camera sensor, so that the light collected at the sensor corresponds to the image a camera would record. In doing so, ray tracing accounts simultaneously for the effects of the optical system and for the effects induced by the refractive-index field in the domain. This unified treatment is particularly well suited to study beam deflections caused by localised refractive-index gradients, which are central to the present problem.

The core of the method is the parallelised integration of individual light beam trajectories by numerically solving the geometric-optics ray equation (Euler–Lagrange equation) through the medium:

9 $$\frac{{\mathrm{d}}}{{{\mathrm{d}}s}}\left( {n\frac{{{\mathrm{d}}\vec{r}}}{{{\mathrm{d}}s}}} \right) = {\mathrm{grad}} n$$

where s is the path length, n is the refractive index, and $\vec{r}\left( s \right)$ is the position along the beam path. While propagation through the inhomogeneous medium is handled by this differential equation, interactions with optical elements, such as mirrors, lenses, windows, and apertures, are treated geometrically: at each intersection. The law of reflection, Snell’s law for refraction, and aperture transmission are applied to update the beam direction.

Once beams have been advanced through the optical path, the algorithm assembles the final image by solving the intersection of each beam with the camera sensor. At this stage, diffraction is incorporated by approximating the Airy pattern with a Gaussian, and the corresponding diffraction diameter is computed from the optical system parameters. In this way, both geometrical and diffraction effects contribute to the simulated sensor irradiance.

The implementation used here follows Rajendran et al. (2019), whose freely available code is tailored to aero-thermodynamic optical simulations. For the present application, the available code has been adapted to run on a Windows platform; therefore, the complete algorithmic details are as reported in Rajendran et al. (2019). The new code, which is compatible with Windows, is available in the repository published alongside this study.

## Results and discussion

### Numerical simulation of the ray bending at refractive-index gradients

The ray-tracing simulation is based on the geometry of the experimental set-up, described above. In this set-up, the screen and camera were positioned at a distance of 0.5 m from the object. As part of the study, three configurations were analysed at ambient pressures of 1 bar, 30 bar, and 60 bar. The medium was air. Under the experimental conditions used here, the combustion zone of the swirl-stabilised flame appears as a torus, with a maximum temperature of 2000 K, which is assumed to be a conservative upper bound for the adiabatic flame temperature for such a lean mixture in all three scenarios. The ambient temperature was set at 400 K at 1 bar, and at 800 K for the other two cases; the increased ambient temperature for the pressurised case is intended to mimic engine-relevant surrounding air conditions. In both the simulation and the experiment, a linear fringe pattern with a period of 8 pixels was used, with the fringe spacing corresponding to a distance of 1 mm. This fringe pattern is used to assess the LOS displacement *Δx*.

**Fig. 4:**
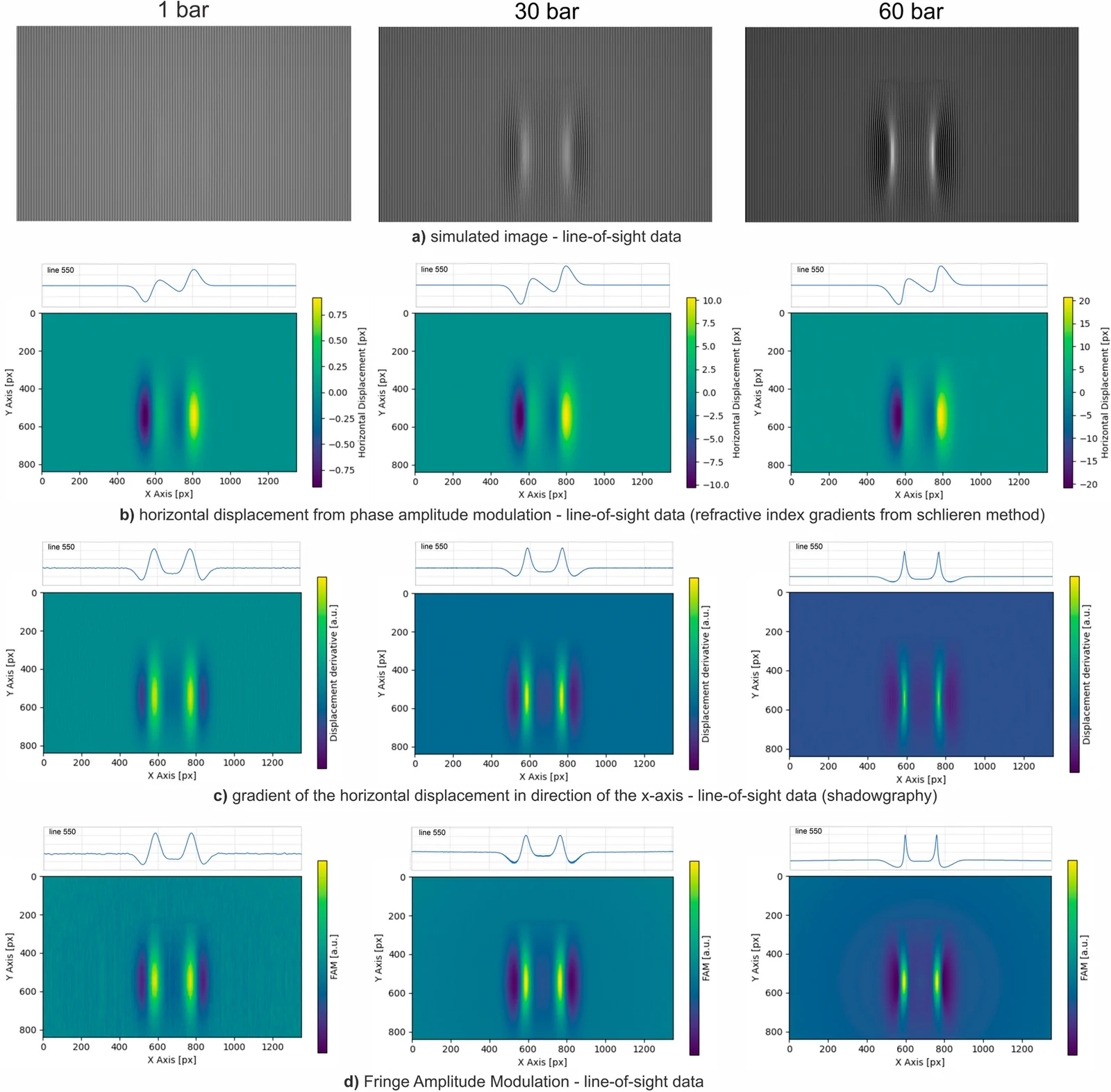
Numerical simulation of three configurations at ambient pressures of 1 bar, 30 bar, and 60 bar. Section **a** presents the simulated images, section **b** displays the horizontal displacement Δx obtained from the HBOS Fourier analysis, section **c** shows the gradient of the displacement Δx from section **b**, and section **d** presents the shadows obtained from FAM analysis. All sections show a horizontal central line through the measured object, along with their relative 2D LOS results

The results of the simulations for the three conditions are displayed in the columns of Fig. 4. As illustrated in Fig. 4a, the first row presents the simulated images of the fringe pattern modulated by the refractive-index gradients. This frequency modulation is clearly visible in the images, as are the shadows caused by the object's beam deflection.

Figure 4b displays the displacement field ${\Delta }x$ obtained from the HBOS Fourier analysis of the fringe pattern in Fig. 4a, and it visualises the LOS gradients of the refractive index as defined in Eq. 1a. Figure 4c is produced by differentiating the *Δx* with respect to *x*, and it presents the corresponding LOS shadow images of the flame. In a linear approximation, these shadows represent the LOS curvature of the refractive-index distribution in the horizontal direction, as described by Eq. 2. The FAM obtained from the Fourier analysis of the simulated fringe pattern is plotted in Fig. 4d. This particular type of shadowgraphy is referred to as 'focussed shadowgraphy', a term introduced by Weinberg (1963). A lively debate on this term appears in Settles (2001). The designation focussed shadowgraphy is somewhat misleading and has consequently been the source of unnecessary controversy. The apparent contradiction arises because focussing is fundamentally incompatible with the principles of shadowgraphy and the schlieren technique when used solely for visualisation. If the schlieren object is brought into focus on the observation plane, the resulting image would cease to be a shadowgram (Settles 2001); only the schlieren would become visible as black-and-white structures in the image. Any other schlieren objects along the LOS that are not in focus would appear as shadows in the image. In contrast to standard schlieren systems, HBOS allows to easily distinguish between refractive-index gradients and shadows by separating the phase and amplitude components of the complex image signal.

In this 'focussed shadowgraphy’, the shadow images are only visible when the camera is not focussed on the schlieren object. In the case under discussion, the focus was set on the background, which was 0.5 m behind the object. This type of shadowgraphy is discussed in several textbooks (Merzkirch 1987; Settles 2001), as well as in technical literature (Winckler 1948). With regard to focussed shadowgraphy, one effect deserves particular attention: the change in depth of field with respect to the aperture value (Settles 2001). As the f-number increases, so does the depth of field. This makes it possible to adjust the sensitivity accordingly in focussed shadowgraphy.

As the f-number is increased, the imaging cone narrows, and the depth of field grows (Hecht 2002). The larger depth of field tends to suppress aberrations of the carrier signal, and local aberration is required before the fringe contrast is perceptibly attenuated. Consequently, low‑gradient regions produce little fringe‑amplitude modulation, while high‑gradient zones, with larger light redistribution, still reduce the carrier contrast and may even suffer ray blockage when the aperture acts as a schlieren stop. In this way, increasing f-number effectively rejects weak, slowly varying curvature and emphasises compact, high‑gradient features.

The following more detailed analysis shows that deviations in the shadow patterns (Fig. 4c versus Fig. 4d) are particularly evident in the 60 bar burner model. Despite the linear approximations for Eqs. 1a and 2 being valid, with a maximum background pattern displacement of 20 pixels corresponding to a confined displacement *Δx* of 2.5 mm at a distance of 0.5 m, the fundamental assumptions for Fourier analysis are no longer valid.

To ensure the accuracy of the heterodyne method in measuring phase and amplitude modulation of the fringes, two points must be strictly observed. Firstly, the carrier frequency of the fringes must be higher than the modulation frequency. This is not the case for the 60 bar simulation. The strong modulation of the carrier frequency observed here results in the first- and second-order modulations overlapping in the frequency domain. Furthermore, in several places, the amplitude of the fringes varies at the same frequency as the underlying harmonic function. In order to avoid such errors, it is necessary to increase the camera resolution in tandem with the frequency of the fringe pattern during the experiment, provided that the field of view is to remain the same. This process should be continued until the modulations of the first- and second-order components no longer overlap in the frequency spectrum.

**Fig. 5:**
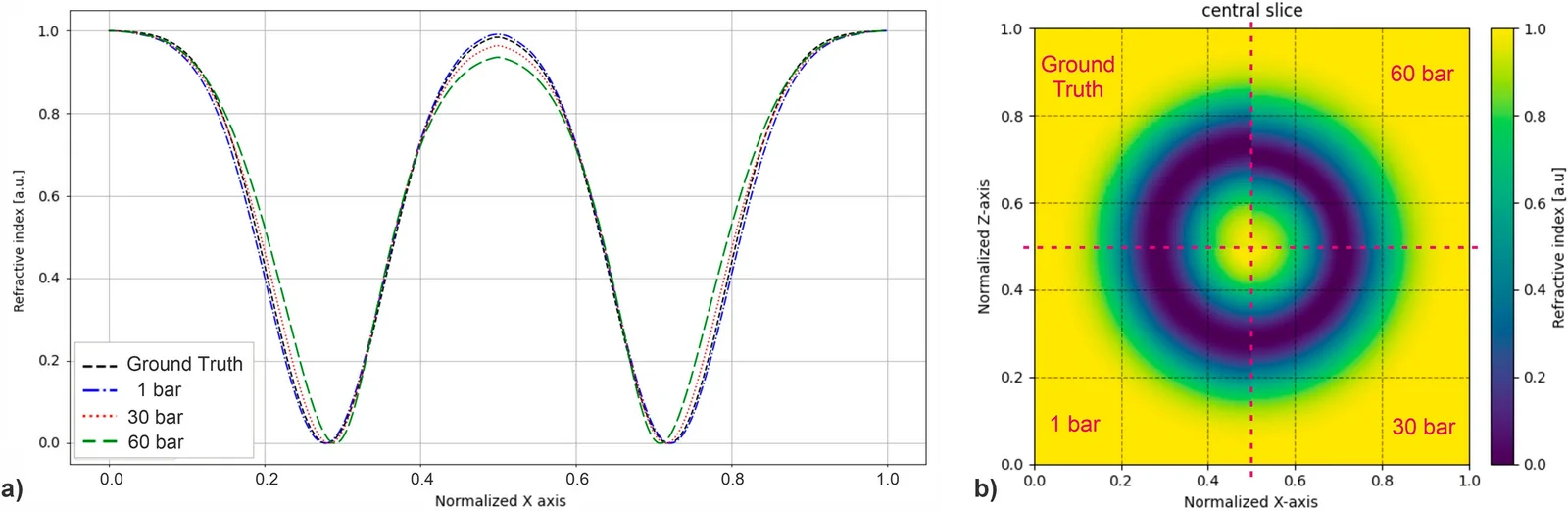
Tomographic reconstruction of the axisymmetric refractive-index field at 1 bar, 30 bar, and 60 bar pressures. This reconstruction was performed on a plane located at the centre of the phantom used in the simulation (ground truth), and then compared to the tomographic reconstructions of the refractive-index gradients (Fig. 4b) obtained from the simulated fringe patterns. Section **a** compares the radial distribution for all three conditions, while section **b** compares the central plane between ground truth (upper left side) and the scenarios with ambient pressure of 1 bar (lower left side), 30 bar (lower right side), and the 60 bar (upper right side) condition

Next, to investigate the influence of the above-mentioned uncertainties on the results, a tomographic reconstruction of the axisymmetric refractive-index field was performed for a plane at the mid-section of the phantom used in the simulation. This was done using the LOS gradients (displacements *Δx* plotted in Fig. 4b) obtained via Fourier analysis. In Fig. 5a, the radial distributions for all three investigated scenarios, obtained via Abel inversion of the ray-traced LOS fields, are compared with the corresponding radial distribution of the phantom (the ground truth). Figure 5b shows a comparison of the reconstructed refractive indices of the plane at 1 bar (on the left) and the same plane at 60 bar (on the right). While the Pearson’s correlation coefficient changes from 0.999 at 1 bar to 0.992 at 60 bar, the root mean squared error changes from 0.0117 to 0.0537, respectively. It is important to note that, although the reconstructed structures remain similar in these local reconstructions, with the 60 bar scenario being flawed due to an incorrectly interpreted fringe pattern, the local absolute values do not.

**Fig. 6:**
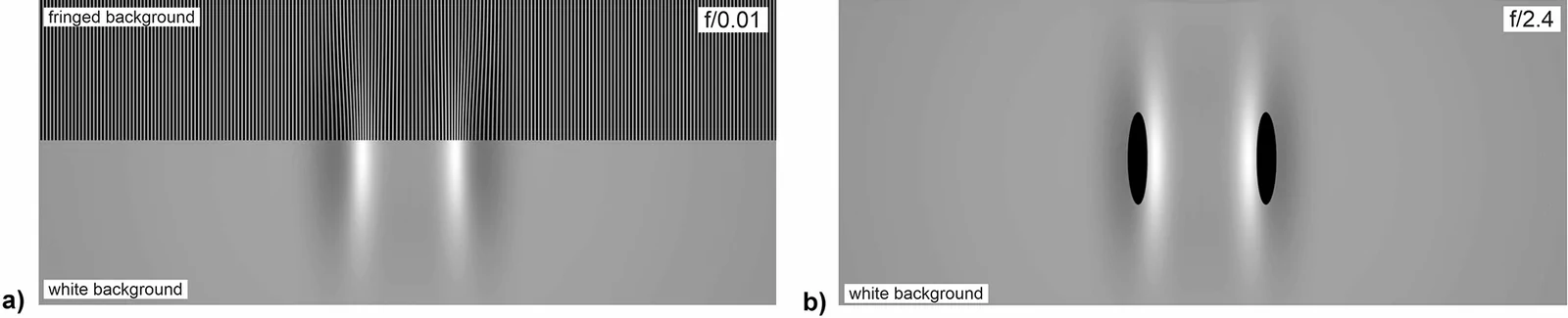
Sections **a** and **b** simulate the effect on the focussed shadowgraphy when different f-numbers for the 30 bar configuration are used while imaging a uniformly bright background. For comparison, a fringe pattern with its modulation is superimposed on the image in section **a**, with a strong gradient evident in the LOS phase modulation of the fringes causing a ray deflection so strong that the camera lens starts acting as a schlieren stop

In the simulation presented in Fig. 4, the camera aperture was set to a f-number of f/0.01. As indicated in Fig. 2a, even a slight deflection of the light rays over a long distance can cause the beam to be blocked by the aperture before it can reach the sensor. For Fig. 6a and b, this effect was numerically simulated for the 30 bar configuration using a white background. In Fig. 6a, an f-number of *f*/0.01 was selected for the simulation, while *f*/2.4 was chosen for Fig. 6b. The darkening of the area behind the torus is caused by the refractive-index field, acting similarly to a diverging lens. As the temperature increases, the density within the torus decreases. Consequently, the strongest outward radial deflection of the rays occurs in the region of the outer refractive-index gradients, as indicated schematically in Fig. 2a. This strong gradient is evident in the LOS phase modulation of the fringes shown in the insert of Fig. 6a. This effect is well documented in the literature, as the camera aperture acts as a schlieren stop (Settles 2001), and is also observed in standard shadowgraphy experiments.

In this set-up, the onset of change in fringe contrast is governed by the aperture (f-number). Under nominal conditions, the relationship is straightforward. At f/2.4, only the 60 bar case, with a maximum fringe displacement of 20 pixels, experiences ray blockage by the aperture, due to the strong deflection of light rays. In contrast, for the 30 bar case blockage by the aperture begins only when the aperture is stopped down from f/2.4 to f/5.6, while the ambient-pressure case shows no ray blockage even at f/8.

The simulations show that smooth ray deflection, which presents frequencies much lower than the carrier signal, linearly widens the bandwidth of fringe modulation for both phase and amplitude (Takeda 1990). In contrast, aperture-driven blockage is nonlinear, generating higher harmonics and spectral broadening that violate the spectral nonoverlap assumption and bias fringe phase and amplitude simultaneously. In the linear, nonray-blocking regime, FAM is a physically meaningful focussed shadowgraph proxy for LOS curvature, with focussing or defocussing also controlled by the aperture.

### Phase and fringe amplitude modulation in a swirl-stabilised flame

A total of 10,000 LOS frames have been acquired and processed with the HBOS algorithm. The experimental results associated with this are discussed here to highlight how the conclusion drawn from the simulation outputs can be used to extract physically meaningful information for a swirl-stabilised turbulent flame.

**Fig. 7:**
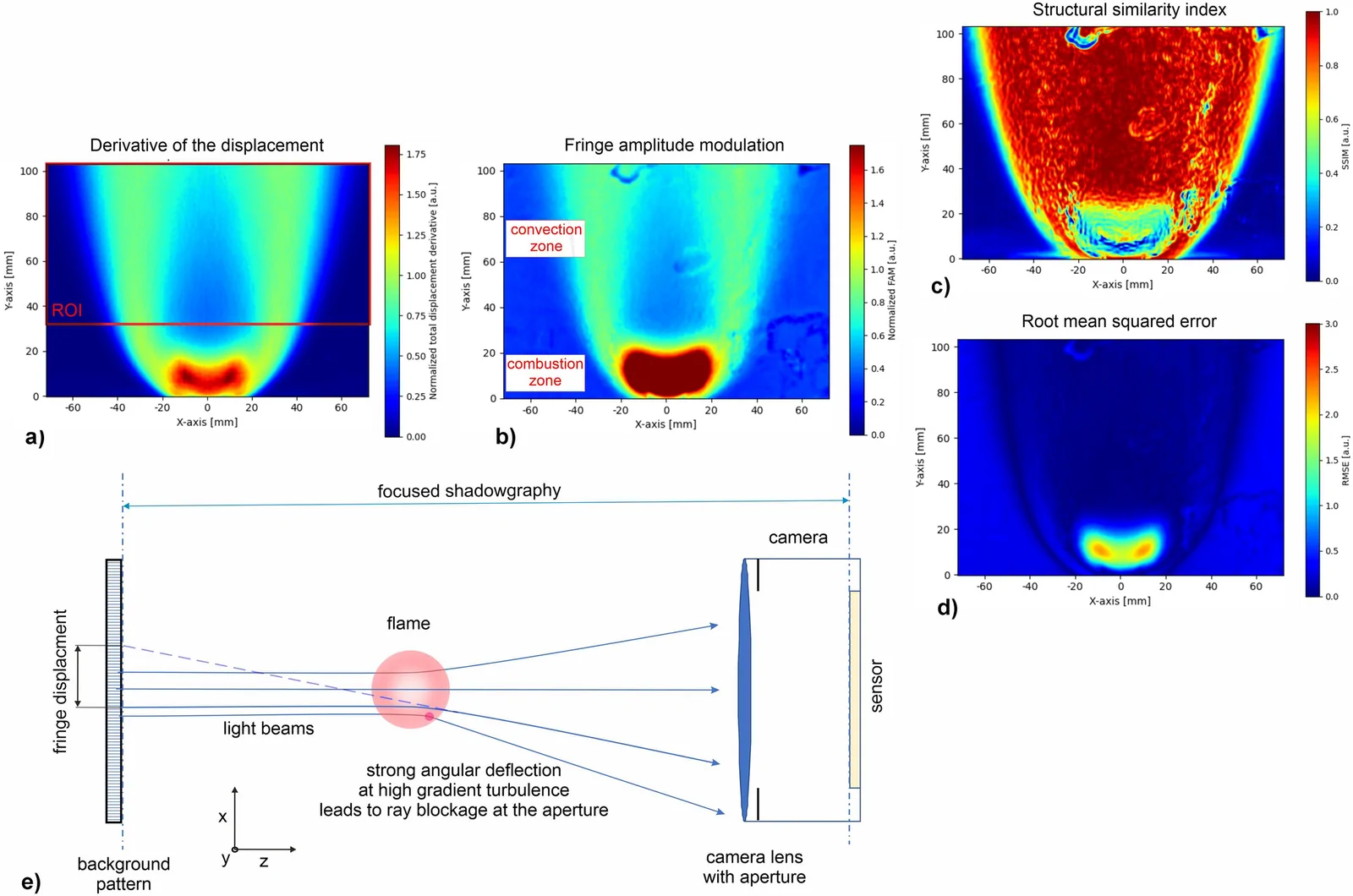
Section **a** and **b** compare the derivative of the total fringe displacement obtained from the fringe phase modulation in x- and y-direction with the fringe amplitude modulation for the methane flame object of the study for an average of 10,000 frames and *f*/5.6. The amplitudes in the whole field have been normalised based on the values within the ROI marked in section **a**. The difference between the two fields is highlighted by the structural similarity index measure (SSIM) in section **c** and the root mean square error (RMSE) in section **d**. Section **e** details the two contributions to the FAM, namely the focussed shadowgraphy and the blockage of light at the aperture in case of strong deflection in the high-gradient turbulent eddies

To further explore the quantitative nature and robustness of the relationship discussed in the previous section, Fig. 7 compares the derivative of the total fringe displacement obtained from the fringe phase modulation in x- and y-direction (in Fig. 7a) with the FAM in Fig. 7b for the methane flame object of the study. In the figures, the amplitudes in the whole field have been normalised based on the values within the ROI marked in Fig. 7a. For each of the two figures, the 10,000 frames were obtained as the Euclidean norm of the two components in the x and y directions, and then averaged. For the recordings, *f*/5.6 was used.

The difference between both fields obtained from the phase and amplitude modulations is highlighted by the calculation of the structural similarity index measure (SSIM) and by the root mean square error (RMSE) between the two fields, shown, respectively, in Fig. 7c and d. In the convection zone, the SSIM signal shows high structural agreement between the two fields, along with a minimum RMSE, indicating strong robustness of the relationship across the phase and amplitude modulations. The combustion zone presents the opposite behaviour. The SSIM map was obtained by comparing Fig. 7a and b with a ROI size of 19 pixels, a Gaussian weighting and an appropriate data range. These observations indicate that the FAM signal in the convection zone behaves as a representation of the second derivative of the refractive index, namely a proxy for the LOS beam curvature associated with the Hessian of the refractive index. A result is also supported by the ray-tracing simulations in the previous section.

Figure 7e distinguishes two aperture-controlled mechanisms that determine FAM amplitude. First, smooth variations in refractive-index curvature (the LOS second derivatives that drive shadowgraph contrast) cause the emerging ray bundle to develop a finite light intensity redistribution. When imaged through a finite aperture, this redistribution appears as a local variation in the carrier-fringe contrast on the sensor. Stopping down the lens increases depth of field and reduces sensitivity to weak light redistributions. As a result, weak, slowly varying curvature in convection zones produces little FAM, while sharper curvature remains visible (Merzkirch 1987; Settles 2001; Weyl 1954; Hecht 2002). Second, in regions with strong gradients, such as the combustion zone, ray deflections can be large enough that part of the ray bundle falls outside the pupil and is blocked. The aperture then acts as a schlieren stop, producing darkening and reducing carrier-fringe contrast (Weinberg 1963; Settles 2001). Increasing the f-number both suppresses contrast variations from small curvature by increasing the depth of field and makes aperture blockage more likely where deflections are strongest. This explains why the FAM is selective for compact, high-gradient features in experiments, while remaining weak in low-gradient regions (Hecht 2002; Goodman 2005).

**Fig. 8:**
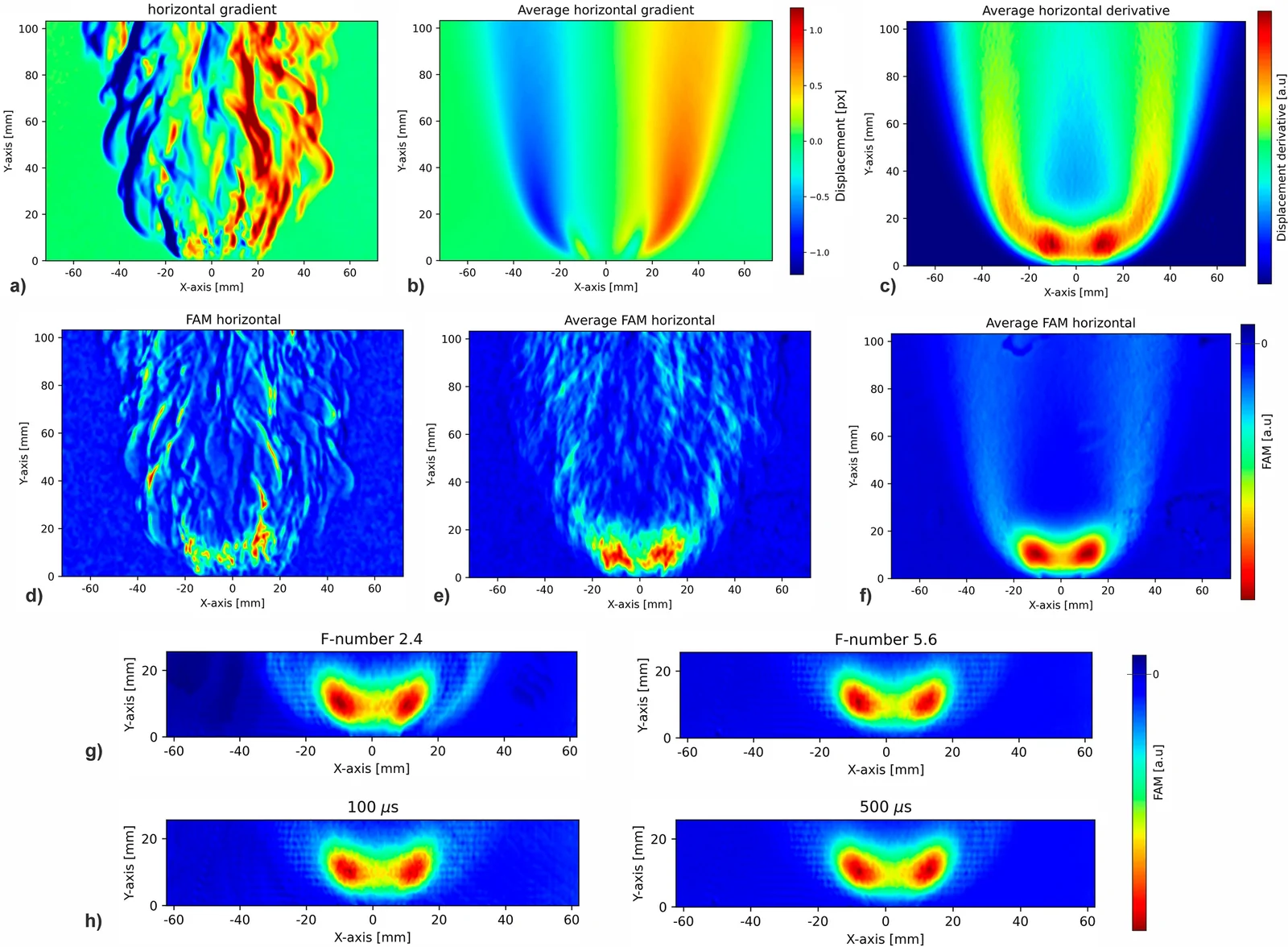
This figure shows different results obtained with the HBOS algorithm when measuring the lean premixed swirl-stabilised methane flame: Section **a** presents an instantaneous displacement signal; Section **b** displays the time-averaged displacement signal over 10,000 frames; Section **c** illustrates the time-average of the absolute value of the horizontal derivative of the phase modulation displacement signal; Section **d** depicts a single frame instantaneous FAM signal; Section **e** features an average over 10 frames from the instantaneous FAM signal; Section **f** provides the time-averaged FAM signal over 10,000 frames; Section **g** compares the time-averaged FAM signals over 1000 frames for aperture *f*/2.4 and aperture *f*/5.6; Section **h** contrasts the time-averaged FAM signals over 1000 frames for an exposure time of 100 µs and an exposure time of 500 µs

To further deepen the discussion, Fig. 8 shows the instantaneous and time-averaged results for both the phase and amplitude modulation fields for the methane flame object of the study. Figure 8a shows the LOS instantaneous displacement *Δx*_*i*_. Figure 8b plots the time-averaged field of this displacement. This effect can be related to the gradient of refractive index in the horizontal direction via Eq. 2. For Fig. 8c, the horizontal derivatives of 10,000 instantaneous frames were calculated. Next, the absolute values of these horizontal derivatives were averaged.

10 $$\overline{{\left| {\frac{{\partial^{2} n}}{{\partial x^{2} }}} \right|}} \approx \mathop \sum \limits_{i} \left| {\frac{{\partial \Delta {\mathrm{x}}_{i} }}{\partial x}} \right|$$

This field can be interpreted as the absolute value of the second derivative of the refractive index in the horizontal direction. Therefore, it is a quantity associated with the LOS curvature of the refractive-index field in the horizontal direction. As also seen in simulations, this curvature field can be interpreted as a numerically derived shadowgraph. It is necessary to notice that, due to the triangular inequality, this averaged field can be obtained only if the average is computed over the absolute value of the instantaneous field.

The second row of Fig. 8 presents the results of the FAM analysis. In Fig. 8d, the instantaneous horizontal FAM field obtained via the HBOS procedure is shown. This field arises solely from changes in the intensity of the pattern in the background perceived by the camera due to the presence of the flame, and it is therefore mathematically decoupled from the phase modulation of the fringe signal. Moreover, under the experimental conditions used here, there is no overlap of the filtered modulation band with the DC component or higher harmonics, as already discussed in Tasmany et al. (2024). Under these conditions, and in the present interpretation from the simulation and in the light of the results discussed in Fig. 7, the fields plotted in Fig. 8d–f exhibit in the convection zone a behaviour similar to that of a shadowgraph: because the object’s refractive properties redistribute light intensity at the sensor, the measurement selectively enhances or suppresses different features depending on the local flow physics.

Building on this interpretation, Fig. 8e reports an average over 10 instantaneous FAM images to illustrate the field’s short-time behaviour. Even with only 10 frames, different behaviour of the convection and combustion zones is observed compared with the field calculated in Fig. 8c. As the temporal window is extended, this separation becomes more pronounced: Fig. 8f shows the FAM signal averaged over 10,000 frames, where the response remains strong in the combustion zone and is nearly extinguished in the convection zone.

The change in amplitude of the FAM signal between the convection and combustion zones arises from two coupled mechanisms controlled by the acquisition aperture discussed above. On the one hand, there are the shadows produced by the diverging beams, which are involved in both convection and combustion, and on the other hand, the aperture acts as a schlieren stop and blocks highly deviated rays, thereby producing dark spots in regions of high-gradient turbulence; hence, on average, only in the combustion zone.

From what was observed in the convection zone, the aperture controls the FAM sensitivity through depth of field. By setting the circle of confusion, the aperture determines how strongly the fringe contrast is attenuated. In regions of high refractive‑index curvature, the local aberration is large, and the signal at the sensor remains sufficient to reduce the carrier-fringe amplitude; in low‑curvature convection regions, the aberration is small, and the attenuation is weak. Additionally, the modulation transfer function (Hecht 2002), which is dependent on spatial frequency, introduces a stronger aberration associated with small-scale refractive-index-gradient structures when these structures are comparable in size to the carrier signal. Such structures are, on average, only found in the combustion zone.

The aperture-dependent aberration is evident in Fig. 8g: when moving from a wider aperture (*f*/2.4) with a smaller depth of focus to a narrower aperture (*f*/5.6) with a larger depth of focus, and stronger effective ray blocking, the contribution from the convection zone nearly disappears, as anticipated by the preceding analysis.

On the other side, in the combustion zone, the effect of the aperture acting as a schlieren stop is enhanced by closing the aperture, increasing the blockage caused by the high-gradient turbulent eddies in the region of combustion.

In order to rule out motion blur as a contributing factor, Fig. 8h further shows that the lower flame region imaged at 100 µs and 500 µs exposure times yields essentially indistinguishable FAM fields, indicating that the relevant structures are effectively frozen over this time and that the measured contrast attenuation is governed by optical aberration rather than by motion blur.

It should be noted that when the camera aperture acts as a schlieren stop, the modulation transfer function of the optical system is low-pass filtered. This means that high gradients are no longer present in the fringe phase modulation. This bias in the refractive-index gradient is observed not only in HBOS but also in BOS when these gradients produce such high beam deflection that the camera aperture acts as a schlieren stop. To avoid such biased results when detecting density gradients, the distance to the flame might be decreased so that the beam displacement at the aperture is smaller than the aperture’s diameter. If this is not possible due to the heat radiation from the flame, a telephoto lens might be used, having a larger aperture at high f-numbers compared to a standard lens.

## Summary and outlook

HBOS, as implemented and analysed in this work, separates phase and amplitude pathways to yield complementary, physically interpretable information on reactive flows: the phase modulation signal recovers LOS displacements linked to refractive-index gradients, while the FAM signal isolates intensity redistribution independent of phase, thereby acting as a focussed shadowgraph of the flow field. In concert with ray-tracing analysis, this decoupling clarifies that the FAM response is governed by aperture-controlled aberration: by setting the circle of confusion (through focus and aperture). In high-gradient regions, the ray deflection might be so strong that the camera’s aperture acts as a schlieren stop, preventing light from reaching the sensor. As a result, the FAM recorded in the convection zone behaves as a focussed shadowgraphy, serving as a contrast-attenuated proxy for LOS curvature. In the combustion zone, the signal is temporarily lost due to aperture blockage, preferentially emphasising high-gradient, small-scale structures associated with the combustion process. The signal at the sensor is also superimposed with enhanced aberrations when the aperture does not block the light rays deflected at the high-gradient, small-scale structures.

Consequently, the aperture-controlled HBOS approach consistently reveals a response that reliably accentuates regions where temperature peaks are sharp and spatial scales are small. This interpretation is supported by controlled comparisons of acquisition parameters: varying exposure from 100 to 500 µs leaving the measured FAM field essentially unchanged, while adjusting the aperture modulates sensitivity and selectivity in accordance with the aberration-driven contrast-attenuation mechanism.

The FAM signal provides a selective, curvature-based representation, while the phase modulation signal offers quantitative access to refractive-index gradients. Because the method is LOS‑based, volumetric characterisation of refractive index or combustion events requires tomographic reconstruction; nevertheless, the demonstrated stability of the FAM indicator and the straightforward acquisition workflow make both instantaneous and time‑averaged analyses feasible for thermoacoustic studies, as experimentally shown in Tasmany et al. (2025a, b) when comparing LOS and multi-directional FAM with chemiluminescence for phase-averaged and time-averaged fields. Finally, for quantitative deployment, care must be taken to avoid spectral overlap between carrier and modulation components by selecting suitable fringe frequencies and camera resolution, and to tune the aperture for the desired trade‑off between depth‑of‑field and ray blockage by the aperture. These practical guidelines ensure accurate phase recovery and controlled FAM selectivity in reactive‑flow measurements.

Future research will focus on the application of HBOS to the industrially relevant confined flame case and on the possibility to calibrate the HBOS fields in order to estimate local combustion-related quantities.

## Supplementary Information

Below is the link to the electronic supplementary material.

Supplementary file 1 (DOCX 6467 kb)

Supplementary file 2 (PDF 830 kb)

## Data Availability

Sequence data that support the findings of this study have been deposited as required by the funding organisations at https://doi.org/10.3217/cvcf2-28b98. The Python routines utilised in this project are also available for download from this repository. This repository comes with a CC-BY4 legal code.
