# Supplementary material for: The nature of phase and amplitude modulation in heterodyne background-oriented schlieren systems
Source: Exp Fluids. 2026 Aug 25;67(10):140. doi: 10.1007/s00348-026-04289-w (PMC13506633; doi:10.1007/s00348-026-04289-w)
Supplement: Supplementary file 1 [file 348_2026_4289_MOESM2_ESM.pdf]

This text supplements the article entitled 'The Nature of Phase and Amplitude Modulation in Heterodyne Background-Oriented Schlieren Systems'.

The first observation of a schliere

According to Toepler (1864), a schliere is defined as a streak-like area within a glass specimen that exhibits a slight deviation from the overall density of the glass mass. This phenomenon gives rise to complications, most notably in the context of the fabrication of high-quality optical instruments, such as telescopes. However, according to the provided description by August Toepler, these density changes are so minute that they remain undetectable to the unassisted eye. In order to facilitate observation of these minute deviations in the optical beam path, he illuminates the glass, which he positions posterior to the imaging lens of long focal length. He then uses the convex lens to capture an image of a brightly lit point, which August Toepler identifies as the light source. The image of this spot is cut with an opaque edge. The slight beam deflection at the schliere, in conjunction with the long focal length of the optics, results in the schliere in the glass appearing bright against a dark background to the observing eye, passing by the sharp edge due to the light being deflected by the schliere. This method was pioneered by Mach and Salcher (1887), who observed compression shocks around fast-flying projectiles for the first time.

Beam deflection at the refractive index gradient field

Intuitive approach

The subsequent section details the deflection of light rays in a refractive index gradient field of a gas or combustion flow. For the purposes of this intuitive derivation, the reader is referred to the textbooks by Oertel and Oertel (1989), Merzkirch (1987) and Settles (2001). As illustrated in Figure 1a, the beam deflection is discussed in relation to the gradient of the refractive index, *n*. The refractive index field is characterised as homogeneous in the direction of the *z*-axis, with an increase in refractive index observed in the direction of the *x*-axis. The consequence of this is a slight deflection of the beam, the angle of which is denoted by ε. As illustrated in Figure 1b, the speed of light *c* is shown to vary due to the changing local refractive index as a function of the radius of curvature *R* on a circular segment with an opening angle $d\alpha$. In the case of a schliere, the angle ε is extremely small.

This change in the phase velocity of light gives rise to the following relationship:

$$\frac{(R+dx)\ d\alpha}{R\ d\alpha} = \frac{(c+dc)}{c} \tag{1}$$

Using the definition of the refractive index $n = \frac{c_0}{c}$, and with c_0 being the vacuum speed of light, we find

$$\frac{dn}{dc} = -c_0\ \frac{1}{c^2} \tag{2}$$

and

$$\frac{dn}{n} = -c_0\ \frac{1}{c^2}\ \frac{dc}{n} = -\frac{dc}{c} \tag{3}$$

Substituted into Equation 1, this yields

$$\frac{dx}{R} = -\frac{dn}{n} \tag{4}$$

or after rewriting the equation

$$\frac{1}{R} = -\frac{1}{n}\frac{dn}{dx} \tag{5}$$

Equation 5 is the foundation for all subsequent considerations. If the absolute value of the refractive index is close to 1, light deflection is proportional to the gradient of the refractive index, with the opposite sign to the curvature of the wavefront. This indicates that ray beams, which are always perpendicular to the wavefront, are deflected towards the regions with a higher refractive index. However, in the case of internal combustion applications operating under high pressure, the absolute value of the refractive index can no longer be considered close to 1. Therefore, the absolute value of the refractive index must be taken into account. Even at the early stages of developing a precise mathematical model for beam deflection in refractive index fields, the term on the right-hand side was denoted by ln n.

Up to now, only local beam deflection has been considered. The next step is to consider the integral beam deflection across the line-of-sight (LOS), as shown in Figure 2. The curvature is defined as the reciprocal of the radius of curvature. When expressed in Cartesian coordinates, for a schliere, the square of the deflection in the *x*-direction is many times smaller than 1 with the light beam propagating in the *z*-axis direction. Consequently, the following approximation applies:

$$\left|\frac{1}{R}\right| = \left|\frac{\frac{d^2x}{dz^2}}{\left(1+\left(\frac{dx}{dz}\right)^2\right)^{3/2}}\right| \approx \left|\frac{d^2x}{dz^2}\right| \tag{6}$$

The relationship between the LOS deflection *QQ**, see Figure 2, of the beam in the *x* and *y* directions is thus as follows:

$$\Delta x = l \tan\varepsilon_x = l\frac{dx}{dz} = l\int_D \frac{d^2x}{dz^2}\,dz = l\int_D \frac{1}{n}\frac{\partial n}{\partial x}\,dz = l\int_D \frac{\partial \ln n}{\partial x}dz \tag{7a}$$

$$\Delta y = l \tan\varepsilon_y = l\frac{dy}{dz} = l\int_D \frac{d^2y}{dz^2}\,dz = l\int_D \frac{1}{n}\frac{\partial n}{\partial y}\,dz = l\int_D \frac{\partial \ln n}{\partial y}dz \tag{7b}$$

Where *D* is the extension of the refractive region in the *z* direction through which light propagates.

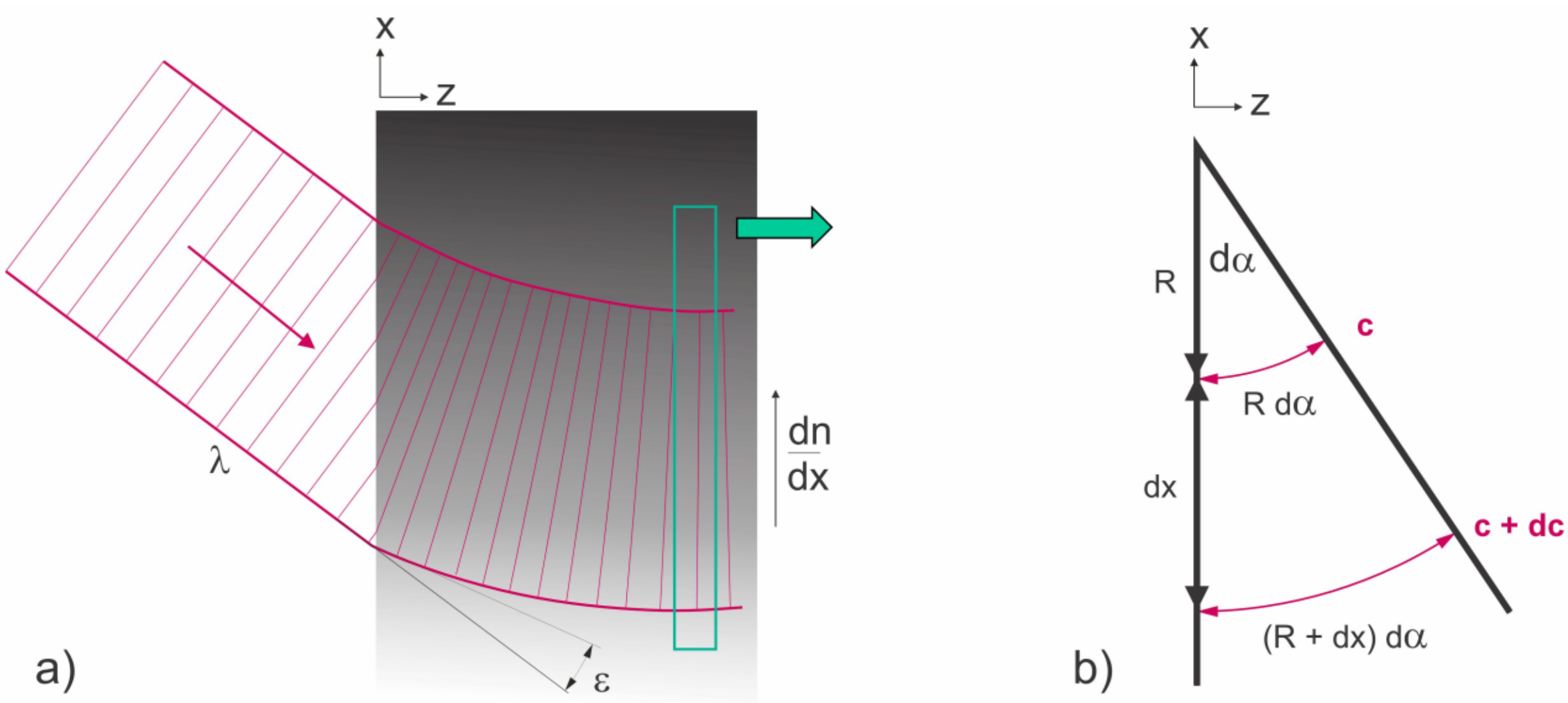

Figure 1 Local beam deflection at the refractive index gradient field of a schlieren. Section a illustrates the deflection. The wavelength of the light wave is denoted by λ, the respective refractive index for that wavelength is labelled n, the coordinates are shown as x and z, and the angle of deflection is indicated by ε. The purely schematic and non-scale representation in section b provides a comprehensive definition of the radius of curvature *R*, the local speed of light *c*, and the opening angle α of a circular segment. Illustration based on Oertel and Oertel (1989).

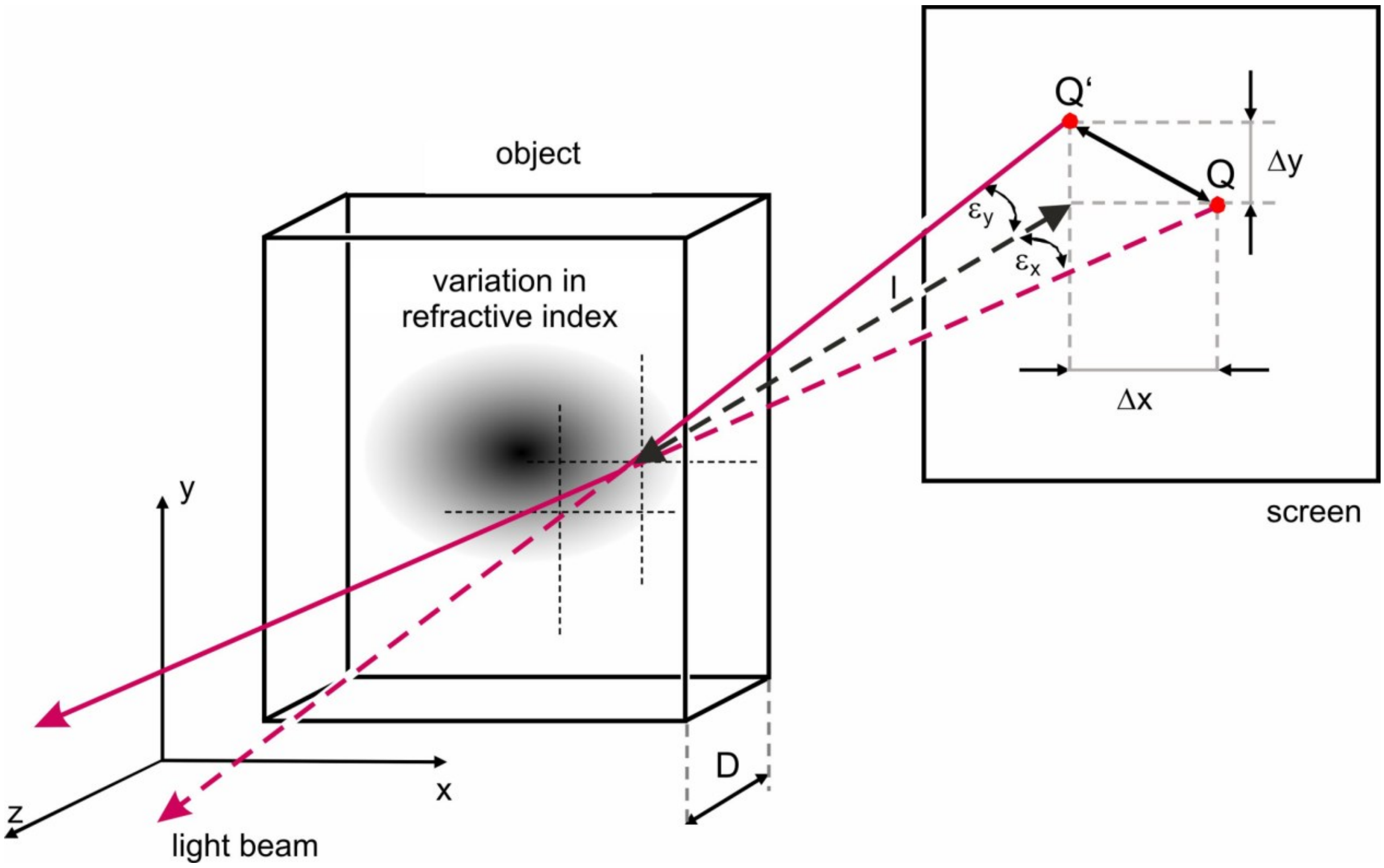

Figure 2 The total beam deflection along the line-of-sight (LOS) is dependent on the object's depth along the z-axis, which is represented by D. The distance between the object and the screen is represented by l. In the context of background-oriented Schlieren methods, any position Q’ on the screen behind the object appears to be shifted by a distance Δx and Δy due to the LOS beam deflection. The illustration is based on Merzkirch (1987). In this figure, the refractive index decreases in a radial direction outwards.

Application of Fermat's principle

In contrast to the previous intuitive approach, Weyl's (1954) analytical approach applies Fermat's law. In accordance with this principle, the optical path length *OPL* for a light ray traversing a refractive index gradient field along the geometrical arc-length parameter *s*, must be stationary relative to infinitesimal variations.

$$OPL = \int n(x, y, z) ds \tag{8}$$

The Euler–Lagrange equation, where $\vec{r}$ is the position vector, determines the actual path of light in inhomogeneous media by minimising the *OPL*. The solution to this variation problem is provided by the corresponding Euler equations in geometrical optics:

$$\frac{d}{ds}\left(n \frac{d\vec{r}}{ds}\right) = grad\ n \tag{9}$$

The arc length parameter *ds* is defined by the Euclidean metric

$$ds = \sqrt{dx^2 + dy^2 + dz^2}\ , \tag{10}$$

and leads to

$$\left(\frac{ds}{dz}\right)^2 = 1 + \left(\frac{dx}{dz}\right)^2 + \left(\frac{dy}{dz}\right)^2\ , \tag{11}$$

and

$$\frac{dz}{ds} = \frac{1}{\sqrt{1+\left(\frac{dx}{dz}\right)^2+\left(\frac{dy}{dz}\right)^2}} \tag{12}$$

Substituting Equation 12 into Equation 9 yields the following equation for the *z*-component

$$\frac{\partial n}{\partial z} = \frac{1}{\sqrt{1+\left(\frac{dx}{dz}\right)^2+\left(\frac{dy}{dz}\right)^2}}\frac{d}{dz}\left(n\frac{1}{\sqrt{1+\left(\frac{dx}{dz}\right)^2+\left(\frac{dy}{dz}\right)^2}}\right) , \tag{13a}$$

and for the *x*-component obtained by expanding with *dx/dz*

$$\frac{\partial n}{\partial x} = \frac{1}{\sqrt{1+\left(\frac{dx}{dz}\right)^2+\left(\frac{dy}{dz}\right)^2}}\frac{d}{dz}\left(n\frac{1}{\sqrt{1+\left(\frac{dx}{dz}\right)^2+\left(\frac{dy}{dz}\right)^2}}\frac{dx}{dz}\right) \tag{13b}$$

In the following calculation, the reciprocal of the root value is substituted by

$$w = \frac{1}{\sqrt{1+\left(\frac{dx}{dz}\right)^2+\left(\frac{dy}{dz}\right)^2}} \tag{14}$$

The derivative in Equations 13a and 13b, therefore, gives us

$$\frac{\partial n}{\partial z} = w\left(w\frac{\partial n}{\partial z}+ n\frac{dw}{dz}\right), \tag{15a}$$

and

$$\frac{\partial n}{\partial x} = w\left(w\frac{\partial n}{\partial z}\frac{dx}{dz}+ n\frac{dw}{dz}\frac{dx}{dz}+n\,w\,\frac{d^2x}{dz^2}\right) \tag{15b}$$

Rearranging Equation 15a gives

$$wn\frac{dw}{dz} = \frac{\partial n}{\partial z} - w^2\frac{\partial n}{\partial z} \tag{16}$$

And substituting Equation 16 into Equation 15b yields

$$\frac{\partial n}{\partial x} = w^2\frac{\partial n}{\partial z}\frac{dx}{dz} + \frac{\partial n}{\partial z}\frac{dx}{dz} - w^2\frac{\partial n}{\partial z}\frac{dx}{dz} + n\,w^2\,\frac{d^2x}{dz^2}, \tag{17}$$

$$\frac{d^2x}{dz^2} = \left(1+\left(\frac{dx}{dz}\right)^2+\left(\frac{dy}{dz}\right)^2\right)\left(\frac{1}{n}\frac{\partial n}{\partial x}-\frac{dx}{dz}\frac{1}{n}\frac{\partial n}{\partial z}\right) \tag{18}$$

And for the *y*-component

$$\frac{d^2y}{dz^2} = \left(1+\left(\frac{dx}{dz}\right)^2+\left(\frac{dy}{dz}\right)^2\right)\left(\frac{1}{n}\frac{\partial n}{\partial y}-\frac{dy}{dz}\frac{1}{n}\frac{\partial n}{\partial z}\right). \tag{19}$$

When the light wave propagates in the direction of the *z*-axis, the deflection in a schlieren is very small, so that the square of the deflection is also negligibly small compared to 1. Since the refractive index gradient is generally of the same order of magnitude in all spatial directions, the simplified system of Equations 7a and 7b results.

$$\Delta x = l\int_D \frac{d^2x}{dz^2}\,dz = l\int_D \frac{1}{n}\frac{\partial n}{\partial x}\,dz \tag{20a}$$

$$\Delta y = l\int_D \frac{d^2y}{dz^2}\,dz = l\int_D \frac{1}{n}\frac{\partial n}{\partial y}\,dz \tag{20b}$$

The shadow of a schliere

Reversing the path of the light rays in Figure 2 produces the result shown in Figure 3. Deflection of the rays at a schliere increases the irradiance at point *Q* and reduces it at point *Q'*. In a first heuristic

approach (Settles 2001), the change in irradiance at a given point on the screen is related to the small LOS deflection of the beam in a given direction (here *x*). With Equation 7a, this intensity change is now proportional to the second derivative of the refractive index along the LOS.

$$\frac{\Delta I}{I'} \approx \frac{\partial \varepsilon_x}{\partial x} \approx \int_D \frac{1}{n} \frac{\partial^2 n}{\partial x^2} \, dz \tag{21}$$

Weyl (1954) provides a more precise approach. Here, a beam of homogeneous intensity *I'(x',y')* passes through the inhomogeneous object and is mapped onto an intensity distribution *I(x, y)*. This calculation must consider how the deflected light rays map the input intensities onto the screen intensity. This process is handled by a Jacobian *J(x',y')*, in which *I(x, y)* represents the irradiance, or power per unit area, of the radiation at the screen or sensor, and *I'(x', y')* represents the input irradiance. The law of conservation of power provides

$$I(x, y) = \frac{I'(x',y')}{|\det J(x',y')|}\,. \tag{22}$$

With the identity matrix and the deflections, the Jacobian reads:

$$J(x', y') = \begin{pmatrix} \frac{\partial x}{\partial x'} & \frac{\partial x}{\partial y'} \\ \frac{\partial y}{\partial x'} & \frac{\partial y}{\partial y'} \end{pmatrix} = \begin{pmatrix} 1 & 0 \\ 0 & 1 \end{pmatrix} + \begin{pmatrix} \frac{\partial \Delta x}{\partial x'} & \frac{\partial \Delta x}{\partial y'} \\ \frac{\partial \Delta y}{\partial x'} & \frac{\partial \Delta y}{\partial y'} \end{pmatrix} = \begin{pmatrix} 1 + \frac{\partial \Delta x}{\partial x'} & \frac{\partial \Delta x}{\partial y'} \\ \frac{\partial \Delta y}{\partial x'} & 1 + \frac{\partial \Delta y}{\partial y'} \end{pmatrix}. \tag{23}$$

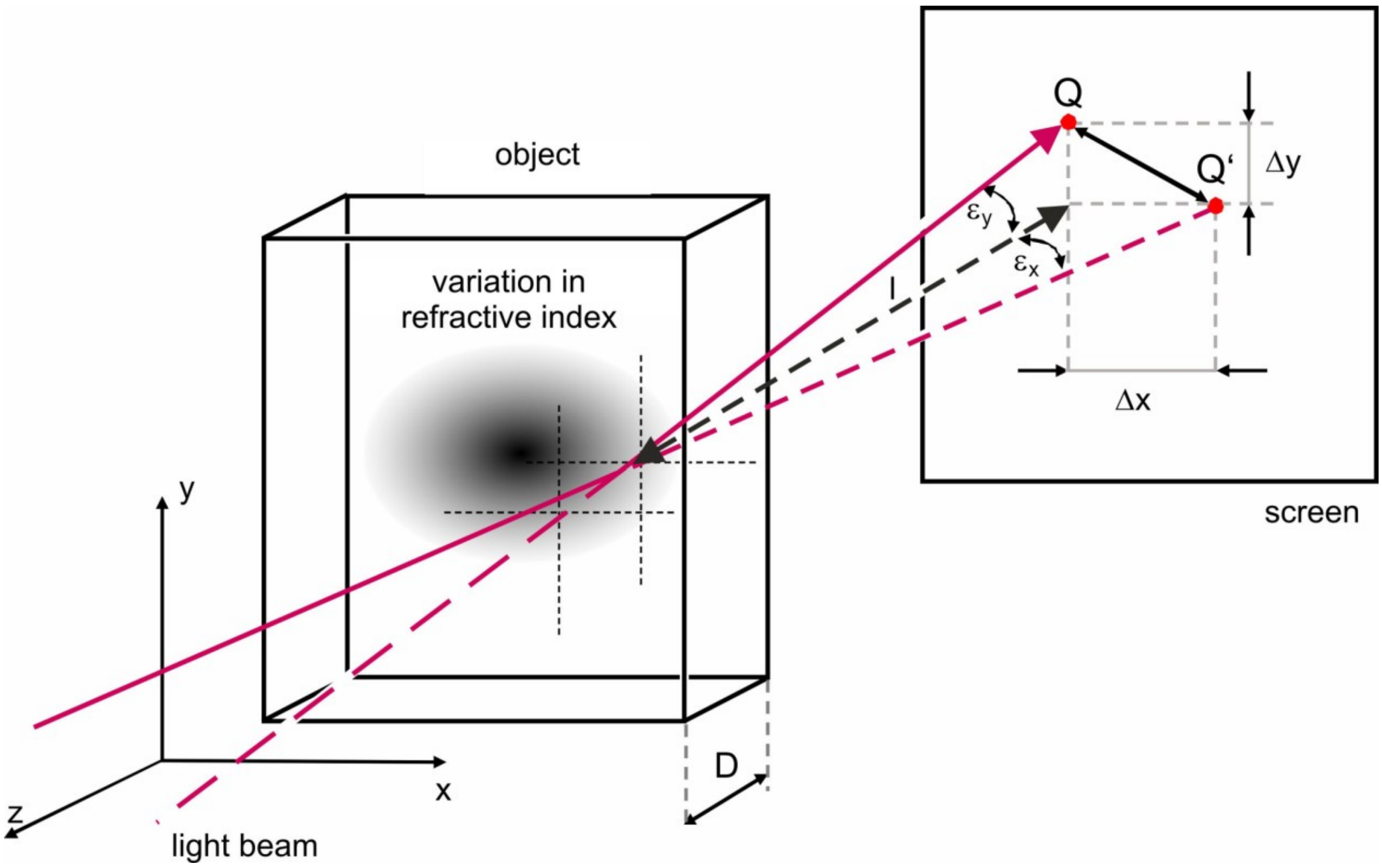

Figure 3 Reversing the beam path in relation to Figure 2 (projection onto the screen) changes the lighting conditions of the screen in the background. While the irradiance increases at point Q, it decreases at point Q'. The illustration is based on Merzkirch (1987). In this figure, the refractive index decreases in a radial direction outwards.

The change in irradiance on the screen is determined solely by the determinant of the Jacobian matrix, provided the irradiance is uniform in the incident parallel beam. When the beam is focused at a single point, the Jacobian operator disappears, and a singularity emerges, with all the energy concentrated at the focal point.

In a shadow image of a schliere, it can be assumed that deflections along the entire beam path from the light source to the screen are minimal. The powers and products can therefore be neglected, resulting in the following linearisation:

$$|\det J(x',y')| = 1 + \ grad \begin{pmatrix} \Delta x \\ \Delta y \end{pmatrix} \tag{24}$$

The correlation between the change in intensity and the beam deflection can be determined following the linearisation process and using the standard definition of shadow contrast (Merzkirch 1987).

$$\frac{I-I'}{I'} = \ grad \begin{pmatrix} \Delta x \\ \Delta y \end{pmatrix} \tag{25}$$

Equations 20a and 20b then yield the following relationship for small beam deflection at a schlieren in a gas flow:

$$\frac{I-I'}{I'} = \ l \int_D \left(\frac{1}{n}\frac{\partial^2 n}{\partial x^2} + \frac{1}{n}\frac{\partial^2 n}{\partial y^2} \right) dz \tag{26}$$

As the distance between the object and the screen increases, so does the contrast.

The shadows at the bottom of a pool

In order to provide the reader with a practical example of shadows induced by transparent media, we report here the following phenomenon, which is experienced in everyday life.

In the 'The Amateur Scientist' column of the scientific journal Scientific American, Walker (1988) provided an illustrative explanation of the shadows cast on the bottom of a pool. When sunlight hits a flat-water surface, the rays are refracted evenly, resulting in uniform illumination of the pool's base. However, if the surface ripples, the waves behave like concave or convex lenses. This results in a significant deflection of the rays due to refraction, as demonstrated in Figure 4.

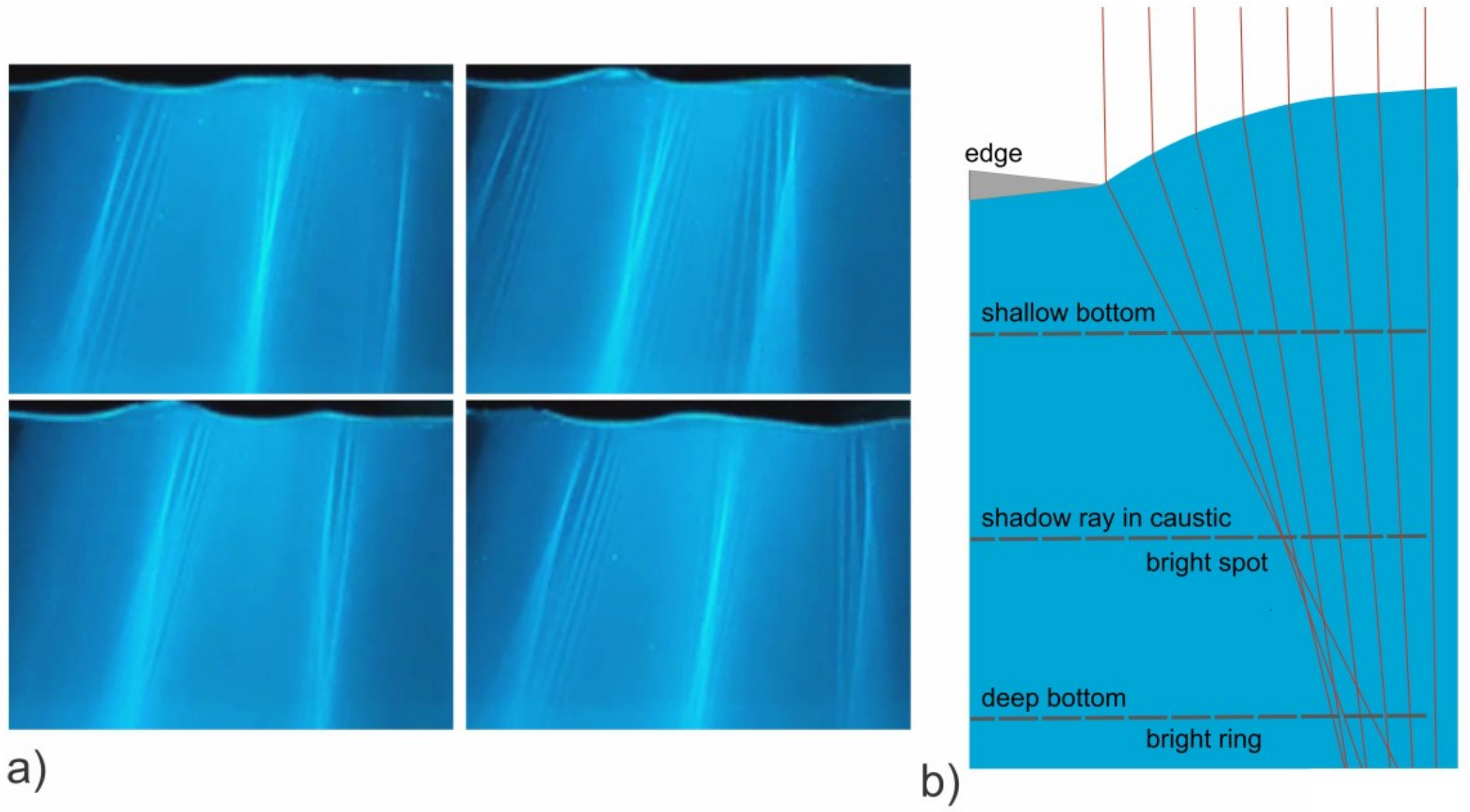

Figure 4 In section a), a laser light sheet is shown cutting through the air-water interface at right angles. Section b) discusses shadows of a sharp edge seen at different depths. Illustration based on Walker (1988).

As demonstrated in Figure 4a, a laser light sheet cuts through the air-water interface at right angles, while Figure 4b discusses shadows seen at different depths. In cases where the water basin is shallow, the shadow of the edge is more or less even, thus showing its position. At a certain critical depth, the shadow creates a bright caustic, remaining at the position of the edge. At a greater depth, the shadow

is no longer representative of the border of the edge, but rather a bright ring of light. To ensure an accurate representation of this scenario using geometrical optics, it is essential to utilise a ray tracing algorithm, whether a commercial one or an open-source option.

Euler-Lagrange equation derivation:

For the sake of completeness and to give the reader a better understanding of the origin of the Euler-Lagrange equation, its derivation is reported here, based on the textbook by Born and Wolf (1980).

Light beams are defined as orthogonal trajectories to the geometrical wavefront, and such a wavefront, which is defined as $S(\vec{r})$, must respect, in the field of geometrical optics, the following constraints:

$$(grad\ S(\vec{r}))^2 = n(\vec{r})^2 \tag{27}$$

With $\vec{r}$ that marks the position vector of a point *P* along a beam trajectory, and n is the refractive index and S is the so-called Eikonal function. The derivation of Equation 27 starting from Maxwell's equations can be found in Born and Wolf (1980).

For the light beams to be locally orthogonal to the wavefront, their local direction has to be coincident with the one pointed by the following vector, $\vec{d}$:

$$\vec{d} = \frac{grad\ S}{n} \tag{28}$$

By defining now $\vec{r}(s)$ as the position of a point *P* on a beam as a function of the length of arc s of its trajectory, it follows that:

$$\frac{d\vec{r}}{ds} = \vec{d} \tag{29}$$

Hence

$$\frac{d\vec{r}}{ds} = \frac{grad\ S}{n} \tag{30}$$

or

$$n\frac{d\vec{r}}{ds} = grad\ S \tag{31}$$

Given the last relation and by applying simple calculus, it is then possible to derive the so-called Euler-Lagrange equation, used in ray tracing.

The first step involves the differentiation of Equation 31 with respect to s:

$$\frac{d}{ds}\left(n\frac{d\vec{r}}{ds}\right) = \frac{d}{ds}(grad\ S) \tag{32}$$

Since S is a function of $\vec{r}$, it is possible to apply the chain rule as:

$$\frac{d}{ds}\big(grad\ S(\vec{r})\big) = \frac{d\vec{r}}{ds}\cdot grad(grad\ S(\vec{r})) \tag{33}$$

and, by using the relation from Equation 30 to substitute $\frac{d\vec{r}}{ds}$:

$$\frac{d}{ds}\left(n\frac{d\vec{r}}{ds}\right) = \frac{1}{n} grad\ S\cdot grad(grad\ S) \tag{34}$$

Given the vectorial identity $grad\ S\cdot grad(grad\ S) = \frac{1}{2}grad[(grad\ S)^2]$ and using Equation 27:

$$\frac{d}{ds}\left(n\frac{d\vec{r}}{ds}\right) = \frac{1}{2n}\cdot grad\ n^2 \tag{35}$$

Since $grad(n^2) = 2n\ grad\ n$ It is then derived that the final form of the Euler-Lagrange equation is:

$$\frac{d}{ds}\left(n\frac{d\vec{r}}{ds}\right) = grad\ n \tag{36}$$

The differential equation just derived is then integrated using numerical methods and serves as the basis for all ray-tracing algorithms.

References

Mach E, Salcher P (1887) Photographische Fixirung der durch Projectile in der Luft eingeleiteten Vorgänge. Sitzungberichte der Akademie der Wissenschaften, XCV Bd., II Abth.:764- 780.

Born M, Wolf E (1999) Principles of Optics: Electromagnetic Theory of Propagation, Interference and Diffraction of Light, 7th ed. Cambridge University Press, Cambridge. ISBN 0521642221; EAN 978-0521642224.

Merzkirch W (1987) Flow Visualization (2nd ed.). Academic Press, Inc., San Diego, ISBN 978-0-12-491351-6

Oertel H, Oertel H Jr (1989) Optische Strömungsmeßtechnik. G. Braun, Karlsruhe, ISBN 3-7650-1012-X

Settles G S (2001) Schlieren and Shadowgraph Techniques, Experimental Fluid Mechanics Series, Springer Berlin, Heidelberg https://doi.org/10.1007/978-3-642-56640-0

Toepler A (1864) Beobachtungen nach einer neuen optischen Methode – Ein Beitrag zur Experimental-Physik. Max Cohen & Sohn, Bonn, Germany

Walker J (1988) Shadows cast on the bottom of a pool. Amateur Scientist, Sci. Am. 259:116-119

Weyl F J (1954) Analysis of Optical Methods. Article A,1, in: Ladenburg R W, Lewis B, Pease R N, Taylor H S, Physical Measurement in Gas Dynamics and Combustion IX, High Speed Aerodynamics and Jet Propulsion, Princeton University Press, Princeton, New Jersey.
